# TPL2 kinase activity is required for *Il1b* transcription during LPS priming but dispensable for NLRP3 inflammasome activation

**DOI:** 10.3389/fimmu.2025.1496613

**Published:** 2025-03-18

**Authors:** Denise L. Fahey, Niki Patel, Wendy T. Watford

**Affiliations:** ^1^ Department of Infectious Diseases, College of Veterinary Medicine, University of Georgia, Athens, GA, United States; ^2^ College of Veterinary Medicine, University of Georgia, Athens, GA, United States

**Keywords:** TPL2, Tpl2 kinase, NLRP3 inflammasome, interferons, type I IFN, IL-1b

## Abstract

The NLRP3 inflammasome complex is an important mechanism for regulating the release of pro-inflammatory cytokines, IL-1β and IL-18, in response to harmful pathogens. Overproduction of pro-inflammatory cytokines has been linked to cryopyrin-associated periodic syndrome, arthritis, and other inflammatory conditions. It has been previously shown that tumor progression locus 2, a serine-threonine kinase, promotes IL-1β synthesis in response to LPS stimulation; however, whether TPL2 kinase activity is required during inflammasome priming to promote *Il1b* mRNA transcription and/or during inflammasome activation for IL-1β secretion remained unknown. In addition, whether elevated type I interferons, a consequence of either *Tpl2* genetic ablation or inhibition of TPL2 kinase activity, decreases IL-1β expression or inflammasome function has not been explored. Using LPS-stimulated primary murine bone marrow-derived macrophages, we determined that TPL2 kinase activity is required for transcription of *Il1b*, but not *Nlrp3*, *Il18*, caspase-1 (*Casp1*), or gasdermin-D (*Gsdmd*) during inflammasome priming. Both *Casp1* and *Gsdmd* mRNA synthesis decreased in the absence of type I interferon signaling, evidence of crosstalk between type I interferons and the inflammasome. Our results demonstrate that TPL2 kinase activity is differentially required for the expression of inflammasome precursor cytokines and components but is dispensable for inflammasome activation. These data provide the foundation for the further exploration of TPL2 kinase inhibitor as a potential therapeutic in inflammatory diseases.

## Introduction

Tumor progression locus 2 (TPL2), also known as MAP3K8 or cancer Osaka thyroid (*Cot*), is a serine-threonine kinase that acts as a regulator of host immune responses (1). It also operates as a scaffolding protein, and TPL2’s kinase function is negatively regulated through its interaction with NF-κB p105 and ABIN2 (2–4). Ligand binding to toll-like receptors (TLRs), TNF receptor, and IL-1 receptors activates the IKK complex, composed of IKKα, IKKβ, and IKKγ (5–11). IKK complex activation leads to the phosphorylation and degradation of NF-κB p105 (12). The degradation of p105 releases TPL2 from its complex, allowing it to be phosphorylated and execute its kinase activity, initiating downstream signaling cascades, such as NF-κB, ERK, JNK, and p38. Through these pathways, TPL2 regulates the production of many pro-inflammatory cytokines, including IL-1β, IL-6, TNF, and IFN-β (2, 6, 13, 14). In addition to its kinase activity, TPL2 regulates the expression of proteins NF-κB p105 and ABIN2 through its scaffolding function (2–4).

The NLRP3 inflammasome is crucial in the innate immune system’s initial sensing of pathogens and has a role in multiple inflammatory diseases, including inflammatory bowel disease (15, 16), atherosclerosis (17, 18), and multiple sclerosis (19, 20). The NLRP3 inflammasome is a complex of NLRP3, ASC, and caspase-1, that functions by cleaving immature pro-inflammatory cytokines, pro-IL-1β and pro-IL-18, as well as pore-forming gasdermin-D (GSDMD) into their active forms (21–24). NLRP3 inflammasome activation occurs via a two-signal process. In priming, or signal 1, microbial components or extracellular cytokines are recognized by cytokine receptors and pattern-recognition receptors (PRRs), such as TLRs and NOD-like receptors. This signaling cascade initiates NF-κB activation, leading to *Nlrp3*, *Il1b*, and *Il18* mRNA expression (25). During activation, or signal 2, the NLRP3 inflammasome protein complex of NLRP3, ASC, and pro-caspase-1 assembles, triggering its catalytic cleavage of caspase-1 (26–29). Additionally, immature pro-IL-1β and pro-IL-18 are cleaved by caspase-1 into biologically active cytokines, IL-1β and IL-18. The N-terminal domain of GSDMD protein is cleaved, creating a pore through which the pro-inflammatory cytokines exit the cell (24, 30–32).

TPL2 is critical for controlling inflammation and host responses, but there is limited knowledge on its regulation of NLRP3 inflammasome function, which is also recognized to be highly regulated by phosphorylation events (33, 34). We previously demonstrated that TPL2 induces *IL1b* mRNA expression (14), and TPL2 has been shown to promote IL-1β secretion in various cell types and contexts (5, 35, 36). Despite the recognition that TPL2 is important for inflammasome pro-inflammatory cytokine synthesis, there is a lack of understanding for how TPL2 regulates the expression of inflammasome components, including NLRP3, caspase-1, and gasdermin-D or whether TPL2 is required for inflammasome function. Additionally, ablating TPL2 increases interferon-β (IFN) production, a type I IFN and a vital pro-inflammatory cytokine that provides protection against viral pathogens (37–40). Type I IFNs can inhibit IL-1β production through multiple cellular mechanisms (41–43). Whether elevated type I IFN signaling contributes to the repression of IL-1β transcription in TPL2-deficient macrophages remains unexplored.

In this study, we aimed to distinguish the roles for TPL2 kinase activity and type I IFNs during inflammasome priming and activation. We found that during inflammasome priming, *Il1b* transcription is regulated primarily by TPL2 kinase activity and is independent of type I IFN signaling. Ablating type I IFN signaling decreased *Il18*, *Casp1*, and *Gsdmd* transcription during inflammasome priming, demonstrating the regulatory differences between TPL2 kinase activity and type I IFNs. Finally, TPL2 kinase activity was dispensable for inflammasome activation and IL-1β secretion when pharmacologically inhibited after priming but prior to inflammasome activation.

## Materials and methods

### Mice

Wildtype (WT) C57BL/6 were purchased from The Jackson Laboratory (JAX strain #000664) and bred in-house. *Tpl2^-/-^* mice backcrossed at least nine generations onto the C57BL/6 WT strain were kindly provided by Dr. Philip Tsichlis (5). *Ifnar1^-/-^* mice (B6.129S2-Ifnar1^tm1Agt/Mmjax; #032045-JAX) were kindly provided by Dr. Biao He. *Tpl2^-/-^* mice were intercrossed with *Ifnar1^-/-^* mice to produce *Tpl2^-/-^Ifnar1^-/-^* mice (44). TPL2 kinase-dead (TPL2-KD) mice with a D270A mutation were generated by Dr. Ali Zarrin (6) and generously provided by Dr. Mark Wilson and Genentech, Inc. Animals were housed in microisolator cages at the University of Georgia Coverdell Rodent Vivarium.

### Bone marrow-derived macrophage culture

Bone marrow-derived macrophages were isolated from the tibias and femurs of age-matched 6–10-week-old male and female wildtype (WT), *Tpl2^-/-^*, TPL2-KD, *Ifnar1^-/-^*, and *Tpl2^-/-^Ifnar1^-/-^* mice. Bone marrow cells were cultured in RPMI-160 medium with glutamine (Mediatech, Inc.), 10% heat-inactivated fetal bovine serum (FBS, Neuromics), penicillin-streptomycin (Mediatech, Inc.), HEPES (VWR Chemicals, LLC), 2-ME (Sigma-Aldrich, Co.), and mouse recombinant M-CSF (10 ng/mL, PeproTech, Inc.). Fresh media and M-CSF were added 4 days after isolation. At day 7 post-isolation, cells were harvested using Cellstripper (Mediatech, Inc.) and seeded at 1 x 10^6^ cells/mL in various formats.

### BMDM stimulation and sample collection

BMDMs were pre-treated with or without TPL2 inhibitor TC-S 7006 (10 μM, Tocris) and left unstimulated or stimulated with 100 ng/mL of lipopolysaccharide (LPS) (*E. coli* 0111:B4, InvivoGen) at the time intervals indicated. For some experiments, ATP (5 mM, MP Biomedicals and InvivoGen) was added 4 hours after LPS stimulation for the stated duration.

### RNA isolation and real time quantitative PCR

Cell supernatants were removed. Cells were collected in TRK lysis buffer, RNA was extracted from the BMDMs using E.Z.N.A. Total RNA Kit I (Omega Bio-Tek, Inc.) and converted into cDNA using High Capacity cDNA Reverse Transcription Kit (Applied Biosystems). The relative gene expression was measured using probes purchased from Applied Biosystems with Sensifast Probe Hi-ROX kit (Meridian Biosciences). Real time quantitative PCR was performed on the QuantStudio3 instrument (Applied Biosystems). Samples were normalized to the actin internal control and the respective wildtype sample for the experiment using the ΔΔCT method. The probes used were: *Il1b* (Mm00434228_m1), *Il18* (Mm00434226_m1), *Nlrp3* (Mm00840904_m1), *Casp1* (Mm00438023), *Gsdmd* (Mm00509958_m1), and *Ifnb* (Mm00439552_s1).

### Cytokine measurement

Cell supernatants were collected for cytokine analysis by ELISA. IL-1β cytokine secretion was detected using Mouse IL-1 beta Uncoated ELISA Kit (Invitrogen). IL-18 cytokine was measured by Mouse IL-18 Uncoated ELISA kit with Plates (Invitrogen). IFN-β cytokine secretion was detected using Rapid bioluminescent murine IFN-β ELISA kit (InvivoGen).

### Statistical analysis

Standard statistical analyses were performed with GraphPad PRISM software version 10.4.1 (627). Individual data points are the average of three biological replicates from a single experiment and the data shown represent the mean ± standard error of mean. Differences between groups were analyzed using two- and one-way ANOVA with Tukey’s *post hoc* test for multiple comparisons and were considered statistically significant if p ≤ 0.05. Further statistical analysis details are provided in figure legends.

## Results

### TPL2 ablation decreases *IL1b* expression during inflammasome priming

**Figure 1:**
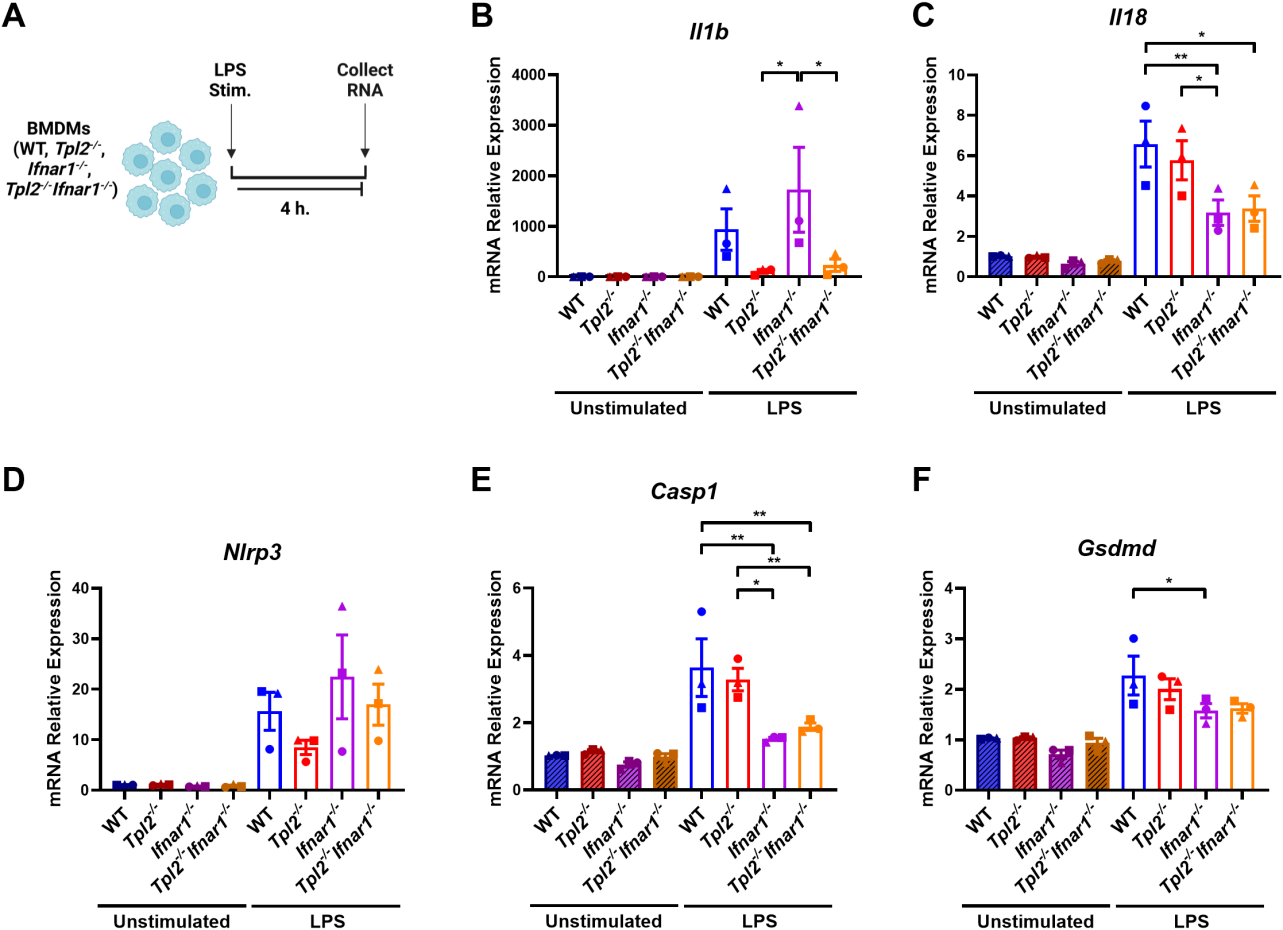
TPL2 ablation decreases *Il1b* expression during inflammasome priming. **(A)** Experimental design depicting BMDM treatment for inflammasome priming. Image created using BioRender. **(B-F)** BMDMs isolated from WT, *Tpl2^-/-^, IFNAR1^-/-^*, and *Tpl2^-/-^IFNAR1^-/-^* mice were either unstimulated or stimulated with 100 ng/mL of LPS (LPS Stim.) for 4 hours. BMDMs were collected for mRNA transcription analysis. Gene expression analysis of *Il1b* **(B)**, *Il18* **(C)**, *Nlrp3* **(D)**, *Casp1* **(E)**, and *Gsdmd* **(F)**. **(B)** Not shown on graph: **p<0.01 unstimulated *IFNAR1^-/-^* vs LPS *IFNAR1^-/-^*. **(C)** Not shown on graph: ***p<0.001 unstimulated wildtype vs LPS wildtype, and unstimulated *Tpl2^-/-^* vs. LPS *Tpl2^-/-^*, and *p<0.05 unstimulated *IFNAR1^-/-^* vs LPS *IFNAR1^-/-^*, unstimulated *Tpl2^-/-^IFNAR1^-/-^* vs LPS *Tpl2^-/-^IFNAR1^-/-^*. **(D)** Not shown on graph: **p<0.01 unstimulated *IFNAR1^-/-^* vs LPS *IFNAR1^-/-^*, and *p<0.05 unstimulated wildtype vs LPS wildtype, unstimulated *Tpl2^-/-^IFNAR1^-/-^* vs LPS *Tpl2^-/-^IFNAR1^-/-^*. **(E)** Not shown on graph: ***p<0.001 unstimulated wildtype vs LPS wildtype, and **p<0.01 unstimulated *Tpl2^-/-^* vs. LPS *Tpl2^-/-^*. **(F)** Not shown on graph: ***p<0.001 for unstimulated wildtype vs. LPS wildtype, **p<0.01 unstimulated *Tpl2^-/-^* vs. LPS *Tpl2^-/-^*, unstimulated *IFNAR1^-/-^* vs LPS *IFNAR1^-/-^*, and *p<0.05 unstimulated *Tpl2^-/-^IFNAR1^-/-^* vs LPS *Tpl2^-/-^IFNAR1^-/-^*. Two-way ANOVA with Tukey’s multiple comparison test was performed. *p<0.05, **p< 0.01. Each data point represents the average of 3 individual mice. Data graphed represent means ± S.E.M. Data are from 3 independent experiments of both male and female mice.

Previous research demonstrated that the absence of TPL2 attenuated IL-1β production during lipopolysaccharide (LPS) stimulation by severely impairing *Il1b* transcription (5, 14, 35, 36). These studies did not distinguish whether TPL2 promotes IL-1β production by solely regulating *Il1b* mRNA synthesis during inflammasome priming or if TPL2 also mediates IL-1β cleavage and secretion during inflammasome activation. To evaluate TPL2’s function during inflammasome priming, bone marrow-derived macrophages (BMDMs) were stimulated with LPS for 4 hours (**Figure 1A**). LPS stimulation caused a trending increase of *Il1b* mRNA expression in wildtype BMDMs (**Figure 1B**). *Tpl2^-/-^* BMDMs stimulated with LPS had reduced induction of *Il1b* mRNA synthesis compared to their unstimulated counterparts (**Figure 1B**).

TPL2 deficiency increases type I IFN cytokine expression, and type I IFNs inhibit IL-1β production (41–43). IFN-β and IFN-α initiate their signaling cascade through the type I IFN receptor, IFNAR1 and IFNAR2, to induce the expression of interferon-stimulated genes (ISGs) (45, 46). Therefore, to test if early type I IFN signaling decreased *Il1b* mRNA synthesis in *Tpl2^-/-^* BMDMs, *Ifnar1^-/-^* and *Tpl2^-/-^Ifnar1^-/-^* BMDMs were simultaneously stimulated with LPS for 4 hours (**Figure 1A**). *Tpl2^-/-^Ifnar1^-/-^* BMDMs lack both TPL2 protein and a functional type I IFN receptor. These BMDMs do not respond to or initiate the type I IFN signaling pathway, but they do produce and secrete type I IFN cytokines (44, 47, 48). Stimulating *Ifnar1^-/-^* BMDMs with LPS induced *Il1b* mRNA levels, similar to LPS-stimulated wildtype BMDMs (**Figure 1B**). If increased type I IFNs were contributing to reduced *Il1b* mRNA synthesis in *Tpl2^-/-^* BMDMs, then *Tpl2^-/-^Ifnar1^-/-^* BMDMs would rescue *Il1b* expression. There was no difference in *Il1b* mRNA expression between LPS-stimulated *Tpl2^-/-^* and *Tpl2^-/-^Ifnar1^-/-^* BMDMs (**Figure 1B**).

In addition to *Il1b*, *Il18* is a pro-inflammatory cytokine precursor produced during inflammasome priming. We examined if TPL2 ablation reduced *Il18* mRNA expression during inflammasome priming by stimulating BMDMs with LPS for 4 hours. There was no difference in *Il18* mRNA expression between wildtype and *Tpl2^-/-^* BMDMs (**Figure 1C**), indicating TPL2 is not required for *Il18* synthesis. Both *Ifnar1^-/-^* and *Tpl2^-/-^Ifnar1^-/-^* BMDMs synthesized lower levels of *Il18* mRNA relative to wildtype BMDMs (**Figure 1C**), consistent with previous publications that observed *Il18* mRNA synthesis is dependent on type I IFNs signaling (49, 50).

Next, we assessed whether TPL2 and type I IFNs altered inflammasome component mRNA synthesis during priming, which could potentially result in modified inflammasome activity. *Tpl2^-/-^* BMDMs have trending decreases in *Nlrp3* mRNA expression (**Figure 1D**). There was no significant difference in *Nlrp3* mRNA levels between wildtype and *Ifnar1^-/-^* or *Tpl2^-/-^* and *Tpl2^-/-^Ifnar1^-/-^* BMDMs (**Figure 1D**). The absence of TPL2 did not alter *Casp1* mRNA expression relative to LPS-stimulated wildtype BMDMs (**Figure 1E**); however, the blockade of type I IFN signaling did significantly lower *Casp1* mRNA synthesis (**Figure 1E**). LPS-stimulated *Ifnar1^-/-^* BMDMs had significantly less *Gsdmd* mRNA expression than wildtype LPS-stimulated BMDMs (**Figure 1F**). Overall, these data indicate that TPL2 deficiency attenuates *Il1b* mRNA expression, while type I IFN signaling blockade decreases *Casp1* and *Gsdmd* mRNA synthesis.

### TPL2 kinase activity regulates *Il1b* mRNA synthesis

**Figure 2:**
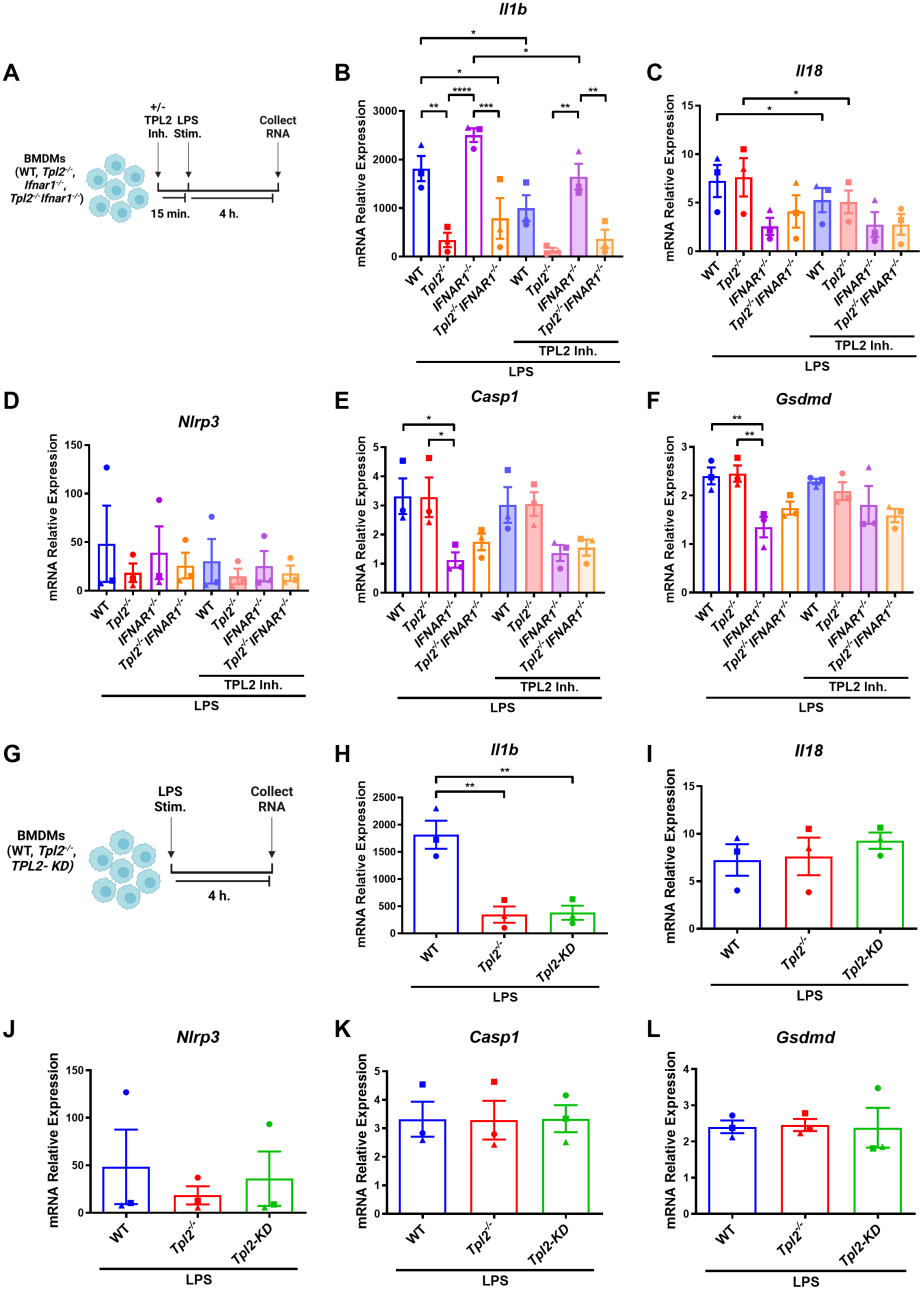
TPL2 kinase activity regulates *Il1b* mRNA synthesis. **(A)** Experimental design depicting BMDM treatment for inflammasome priming with TPL2 inhibitor treatment. Image created using BioRender. **(B-F)** BMDMs isolated from WT, *Tpl2^-/-^, IFNAR1^-/-^*, and *Tpl2^-/-^IFNAR1^-/-^* mice were treated with or without 10 μM of TPL2 inhibitor TC-S 7006 (+/- TPL2 Inh.) for 15 minutes prior LPS stimulation (LPS Stim.). Approximately 4 hours after LPS stimulation, BMDMs were collected for mRNA transcription analysis. Gene expression analysis of *Il1b* **(B)**, *Il18* **(C)**, *Nlrp3* **(D)**, *Casp1* **(E)**, and *Gsdmd* **(F)**. Two-way ANOVA with Tukey’s multiple comparison test was performed. *p<0.05, **p< 0.01, ***p<0.001, ****p<0.0001. **(G)** Experimental design depicting BMDM treatment for inflammasome priming with *TPL2-KD* mice. Image created using BioRender. **(H-L)** BMDMs isolated from WT, *Tpl2^-/-^*, and *Tpl2-KD* mice were stimulated with 100 ng/mL of LPS (LPS Stim.) for 4 hours, then cells were collected for mRNA expression analysis. mRNA expression analysis of *Il1b* **(H)**, *Il18* **(I)**, *Nlrp3* **(J)**, *Casp1* **(K)**, and *Gsdmd* **(L)**. Expression values from WT and *Tpl2^-/-^* BMDMs from the same experiment, also shown in **(B-F)**, are replotted here for comparison. Two-way ANOVA with Tukey’s multiple comparison test was performed. **p< 0.01. Each data point represents the average of 3 individual mice. Data graphed represent means ± S.E.M. Data are from 3 independent experiments of both male and female mice.

TPL2 has dual roles as both a scaffolding protein and a kinase. In its scaffolding function, TPL2 regulates the maintenance of ABIN2 and NF-κB1 p105 proteins; the interaction with these two proteins inhibits TPL2 kinase activity (2–4). To determine if the changes in *Tpl2^-/-^* BMDM *Il1b* mRNA transcription during inflammasome priming were attributed to TPL2’s kinase activity, BMDMs were treated with TPL2 inhibitor 15 minutes prior to LPS stimulation (**Figure 2A**). TPL2 inhibitor treatment significantly decreased *Il1b* mRNA synthesis in wildtype and *Ifnar1^-/-^* BMDMs relative to their LPS-stimulated counterparts, confirming that TPL2 kinase activity promotes *Il1b* mRNA transcription (**Figure 2B**). *Il1b* levels were unchanged in *Tpl2^-/-^* and *Tpl2^-/-^Ifnar1^-/-^* BMDMs treated with TPL2 inhibitor (**Figure 2B**). Wildtype BMDMs treated with TPL2 inhibitor showed a modest reduction in *Il18* mRNA transcription compared to those stimulated with LPS alone (**Figure 2C**). The addition of TPL2 inhibitor did not alter *Nlrp3*, *Casp1*, or *Gsdmd* mRNA synthesis (**Figures 2D-F**).

Because pharmacological inhibition can potentially cause off-target effects, we further verified the importance of TPL2 kinase activity in *Il1b* mRNA production during inflammasome priming. We utilized BMDMs from TPL2 kinase dead (TPL2-KD, *Tpl2^D270A^*) mice, in which the TPL2 protein remains intact but has no kinase activity, to evaluate mRNA synthesis (**Figure 2G**) (6, 51). Unstimulated TPL2-KD BMDMs exhibited no difference in mRNA expression of inflammasome pro-inflammatory precursors or components relative to all genotypes (**Supplementary Figures 1A–E**). LPS-stimulated *Tpl2^-/-^* and TPL2-KD BMDMs expressed significantly decreased *Il1b* mRNA compared to LPS-stimulated wildtype BMDMs (**Figure 2H**). There was no change in *Nlrp3*, *Il18*, *Casp1*, or *Gsdmd* mRNA synthesis between LPS-stimulated wildtype, *Tpl2^-/-^*, and TPL2-KD BMDMs (**Figures 2I–L**).

### Type I IFNs do not suppress LPS-induced *Il1b* transcription but do promote inflammasome component expression

**Figure 3:**
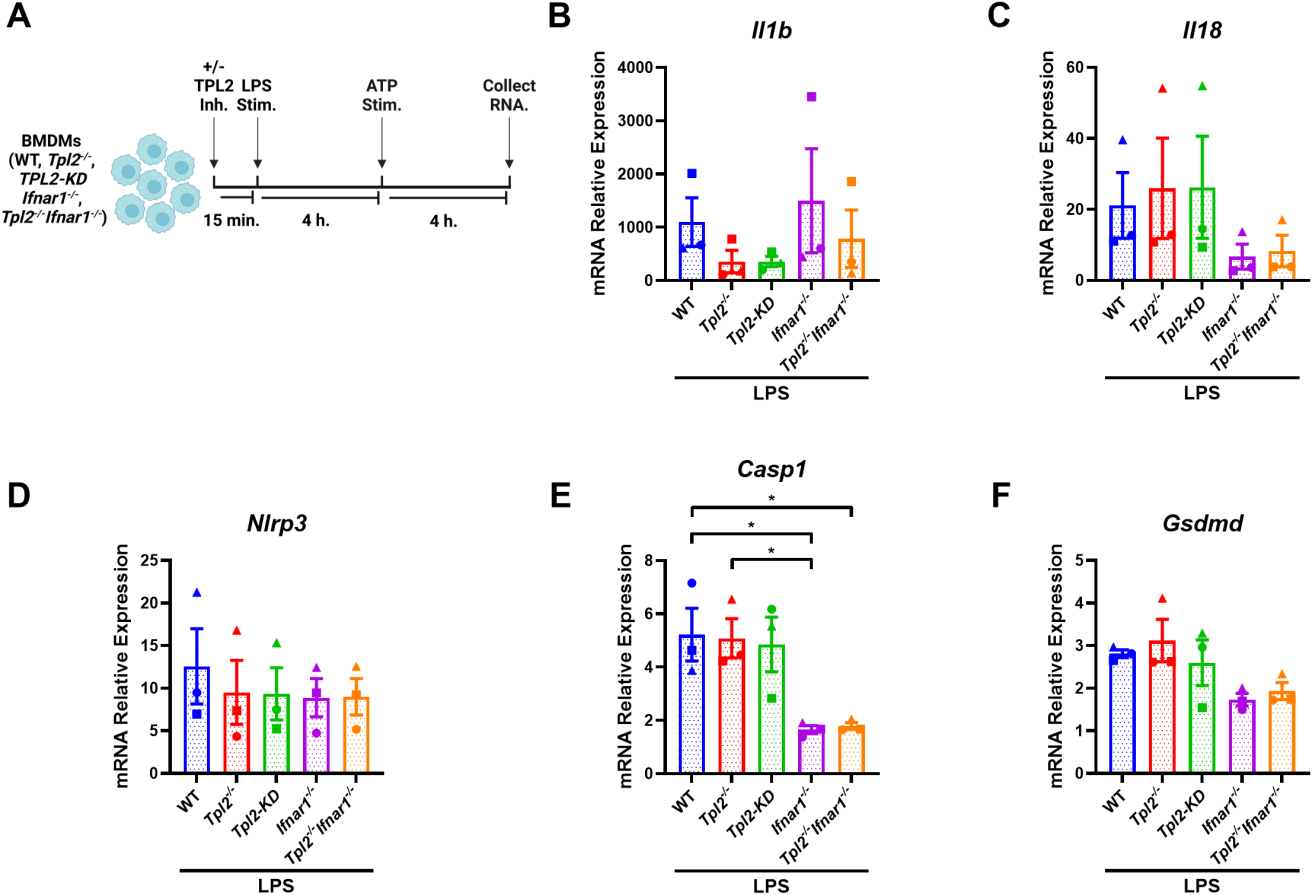
Type I IFNs do not suppress LPS-induced *Il1b* transcription but do promote inflammasome component expression. **(A)** Experimental design depicting BMDM treatment for type I IFN signaling on inflammasome function. Image created using BioRender. **(B-F)** BMDMs isolated from WT, *Tpl2^-/-^, IFNAR1^-/-^*, and *Tpl2^-/-^IFNAR1^-/-^* mice were stimulated with 100 ng/mL of LPS (LPS Stim.) for 4 hours. After 4 hours of LPS stimulation, 5 mM of ATP (ATP Stim.) was added for 4 hours. At the experimental endpoint, BMDMs were collected for mRNA expression analysis. mRNA expression analysis of *Il1b* **(B)**, *Il18* **(C)**, *Nlrp3* **(D)**, *Casp1* **(E)**, and *Gsdmd* **(F)**. One-way ANOVA with Tukey’s multiple comparison test was performed. *p< 0.05. Each data point represents the average of 2-3 individual mice. Data graphed represent means ± S.E.M. Data are from 3 independent experiments of both male and female mice.

Having established the specific role of TPL2 kinase activity during LPS inflammasome priming, we next examined whether increased type I IFN signaling in *Tpl2^-/-^* BMDMs was a contributing factor to alterations in inflammasome mRNA synthesis. *Ifnb* mRNA synthesis peaks 4 hours after LPS stimulation (**Supplementary Figure 2A**), and *Tpl2^-/-^* BMDMs secrete elevated IFN-β relative to LPS-stimulated wildtype BMDMs (**Supplementary Figure 2G**). Additionally, mRNA expression of inflammasome-processed cytokines (*Il1b* and *Il18*) and components (*Nlrp3*, *Casp1*, and *Gsdmd*) over a 24-hour period indicated that transcription occurred by 4 hours of LPS stimulation and often had the greatest synthesis levels at 8 hours (**Supplementary Figures 2B–F**). Therefore, to evaluate the effects of type I IFNs on the transcription of inflammasome components, BMDMs were LPS-stimulated for 8 hours with ATP addition after 4 hours (**Figure 3A**). *Il1b* mRNA levels 8 hours after LPS treatment followed a similar expression trend across the different BMDM genotypes found in **Figure 2B** (**Figure 3C**), and IL-1β cytokine secretion from the various BMDM genotypes matched their *Il1b* mRNA expression (**Supplementary Figure 3A**). *Nlrp3* mRNA expression was not altered by type I IFN signaling (**Figure 3D**). Wildtype and *Tpl2^-/-^* BMDMs expressed significantly higher *Casp1* mRNA relative to BMDMs that lack type I IFN receptors (**Figure 3E**). Both *Ifnar1^-/-^* and *Tpl2^-/-^Ifnar1^-/-^* BMDMs have trending decreases in *Il18* and *Gsdmd* mRNA expression compared to wildtype, *Tpl2^-/-^*, and TPL2-KD BMDMs (**Figures 3C, F**). There was no difference in IL-18 secretion in BMDMs stimulated under these conditions (**Supplementary Figures 4B**). These data indicate that type I IFNs do not contribute to decreased *Il1b* mRNA expression during inflammasome function; however, type I IFNs do promote the expression of inflammasome components, such as *Casp1*.

### IL-1β secretion and inflammasome activation are not dependent on TPL2 kinase activity

**Figure 4:**
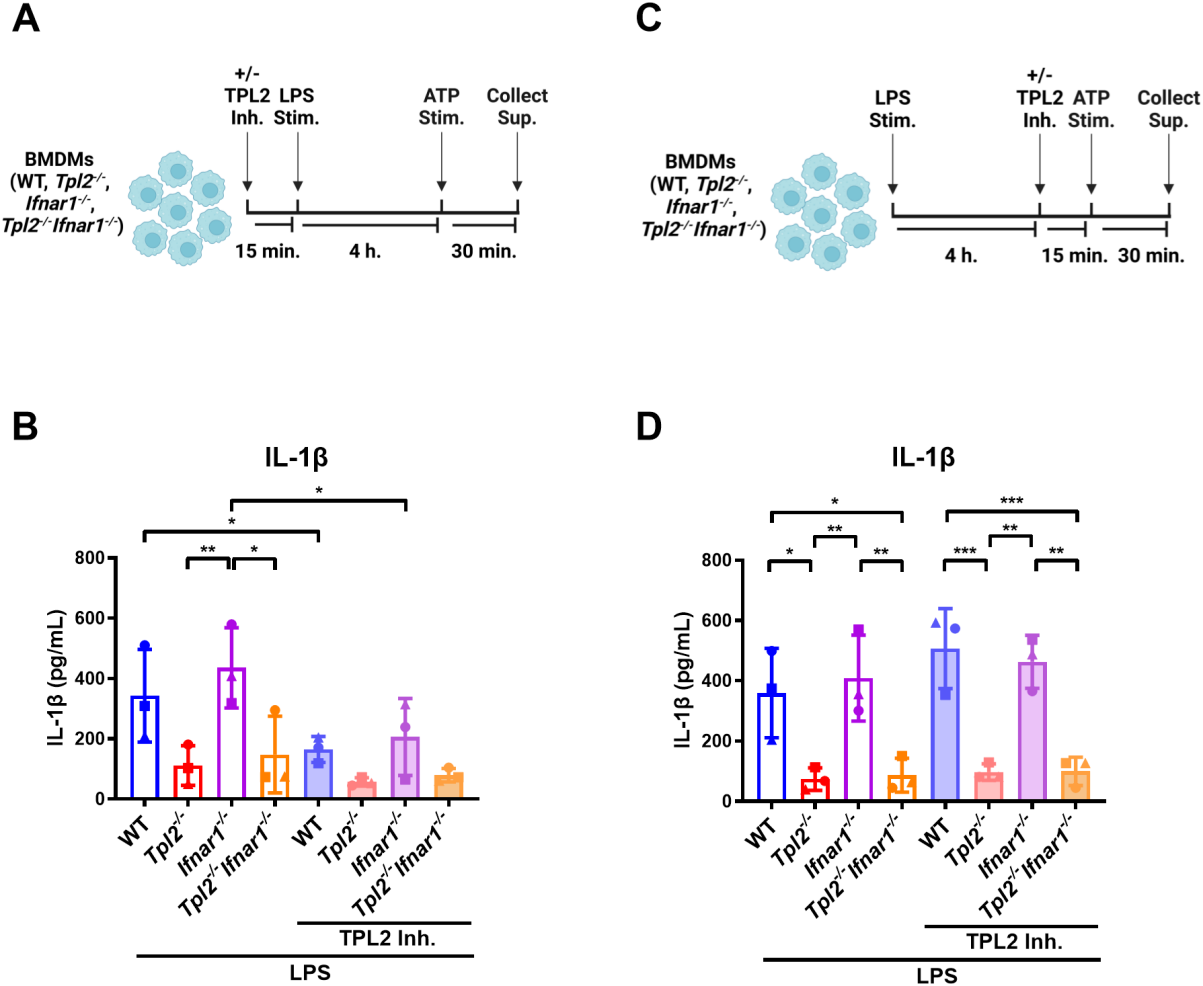
IL-1β secretion and inflammasome activation are not dependent on TPL2 kinase activity. **(A)** Experimental design depicting the role of TPL2 inhibition during inflammasome priming on IL-1β secretion. Image created using BioRender. **(B)** BMDMs isolated from WT, *Tpl2^-/-^*, *IFNAR1^-/-^*, and *Tpl2^-/-^IFNAR1^-/-^* mice were treated with and without 10 μM of TPL2 inhibitor (+/- TPL2 Inh.) for 15 minutes prior to LPS stimulation (LPS Stim.). After 4 hours of LPS stimulation, 5 mM of ATP (ATP Stim.) was added for 30 minutes and supernatant was collected to perform an IL-1β ELISA. **(C)** Experimental design depicting the role of TPL2 inhibition during inflammasome activation on IL-1β secretion. Image created using BioRender. **(D)** WT, *Tpl2^-/-^*, *IFNAR1^-/-^*, and *Tpl2^-/-^IFNAR1^-/-^* BMDMs were stimulated with LPS (LPS Stim.) for 4 hours, then 10 μM of TPL2 inhibitor (+/- TPL2 inh.) was added. 15 minutes after TPL2 inhibitor was added, 5 mM of ATP (ATP Stim.) was added for 30 minutes and supernatant was collected to measure IL-1β secretion by ELISA. Two-way ANOVA with Tukey’s multiple comparison test was performed. *p<0.05, **p< 0.01, ***p<0.001. Each data point represents the average of 3 individual mice. Data graphed represent means ± S.E.M. Data are from 3 independent experiments of both male and female mice.

We have demonstrated that TPL2 kinase activity regulates *Il1b* mRNA expression during inflammasome priming; however, it is unclear if TPL2 kinase activity also affects inflammasome activation and IL-1β release. First, to evaluate the role of TPL2 in inflammasome priming, BMDMs were treated with TPL2 inhibitor 15 minutes prior to LPS stimulation (**Figure 4A**). Four hours later, inflammasome activation was initiated by ATP stimulation for 30 minutes (**Figure 4A**). Wildtype and *Ifnar1^-/-^* BMDMs treated with TPL2 inhibitor before inflammasome priming secreted significantly less IL-1β than their LPS-stimulated counterparts (**Figure 4B**). To assess the effect of TPL2 inhibition on inflammasome activation directly, BMDMs were stimulated with LPS for 4 hours, then treated with TPL2 inhibitor 15 minutes prior to ATP stimulation (**Figure 4C**). LPS-stimulated wildtype and *Ifnar1^-/-^* BMDMs treated with TPL2 inhibitor just prior to inflammasome activation exhibited no reduction in IL-1β secretion (**Figure 4D**), indicating that pro-IL-1β processing by the inflammasome and secretion are independent of TPL2 kinase activity. Overall, these data suggest that TPL2 kinase activity is crucial for *Il1b* mRNA transcription during inflammasome priming but is dispensable for inflammasome activation and IL-1β secretion.

## Discussion

It was previously known that IL-1β production was impaired in response to LPS when TPL2 was absent, but how TPL2 regulated other components of the inflammasome and inflammasome activation remained unclear. Our experiments reveal that TPL2 kinase activity inhibition, either pharmacologically or genetically, impaired LPS-induced *Il1b* mRNA synthesis. In contrast, *Il18*, *Casp1*, and *Gsdmd* transcription are independent of TPL2 but dependent on type I IFN signaling during inflammasome priming. Furthermore, TPL2 kinase activity is critical during inflammasome priming but is dispensable during inflammasome activation and IL-1β secretion.

The absence of TPL2 causes elevated IFN-β expression (37, 38). Though it is well-established that TPL2 regulates IL-1β production, whether the increased type I IFN signaling that accompanies TPL2 ablation contributes to lower IL-1β levels has remained a standing question. To our knowledge, this is the first study that has evaluated both TPL2 and type I IFN signaling in the context of the inflammasome. Our data shows that type I IFN signaling does not contribute to lower *Il1b* expression during inflammasome priming (**Figure 3B**).

In addition to determining the role of TPL2 and type I IFNs on *Il1b*, we also further clarified their roles on inflammasome components during priming. *Casp1* had significantly lower mRNA transcription in *Ifnar1^-/-^* and *Tpl2^-/-^Ifnar1^-/-^* BMDMs compared to wildtype BMDMs (**Figure 1E**). Conflicting reports have suggested that caspase-1 expression is either unchanged or increased after stimulation with type I IFNs and LPS during inflammasome priming (41, 42). Our data aligns with recent studies showing that type I IFN signaling promotes *Casp1* mRNA expression (41, 52–54). LPS-stimulated *Ifnar1^-/-^* BMDMs synthesized significantly reduced *Gsdmd* mRNA relative to LPS-stimulated wildtype BMDMs (**Figures 1F**, **2F**). The regulation of *Gsdmd* by type I IFNs is also in agreement with other previously published studies (41, 52). Our study also helps to clarify conflicting data regarding the role of TPL2 on *Casp1* (35, 36, 54). We demonstrate that TPL2 does not regulate *Casp1* or *Gsdmd* transcription, which is primarily responsive to type I IFN signaling.

While NF-κB contributes to both IL-1β and IL-18 production, these pro-inflammatory cytokines are regulated via different mechanisms. *Il1b* mRNA transcription must be induced by a pro-inflammatory signal or pathogen, and transcription is immediately increased in response to stimulation (55, 56). Conversely, *Il18* is constitutively expressed at steady state (55, 56). Limited evidence indicates that ablating TPL2 kinase activity in human monocyte-derived macrophages and retinal pigment epithelial cells reduces IL-18 secretion in early inflammasome priming (35, 36). While we also found that treating wildtype BMDMs with TPL2 inhibitor modestly reduced *Il18* mRNA expression (**Figure 2C**), there was no difference in *Il18* expression in *Tpl2^-/-^* and TPL2-KD BMDMs relative to wildtype BMDMs (**Figures 2C, I**), suggesting that TPL2 is not a dominant regulatory factor for *Il18*. In murine BMDMs, type I IFN signaling is needed in conjunction with LPS stimulation for *Il18* transcriptional induction, resulting in delayed *Il18* synthesis relative to *Il1b* (49). We observed that both wildtype and *Tpl2^-/-^* BMDMs have a higher average level of *Il18* after 8 hours of LPS, a time when IFN-β secretion is continuing to increase (**Supplementary Figure 2G**). Our data demonstrate that type I IFN signaling is an integral component of *Il18* transcription, while *Il1b* mRNA synthesis requires TPL2 kinase activity (**Figures 1B, C**, **2B, C, H, I**). The different regulatory mechanisms noted above likely account for the differences in TPL2 dependency observed between *Il1b* and *Il18* expression in our study. Despite evidence that type I IFNs promote inflammasome component mRNA synthesis at 4 and 8 hours of stimulation, we did not observe a reduction in IL-1β or IL-18 secretion from *Ifnar1^-/-^* BMDMs at 8 hours (**Supplementary Figures 3A, B**).

In terms of clinical application, TPL2 kinase inhibition has not yet been approved for therapeutic use (57). Our data further clarify how TPL2 kinase activity contributes to inflammation by limiting *Il1b* transcription. Previous studies have shown that TPL2 kinase activity promotes pro-inflammatory cytokine production in both murine and human monocytes and neutrophils (6). The loss of TPL2 kinase activity reduced inflammation and pathogenesis in murine models of arthritis, colitis, and tauopathy (6, 51). In specific contexts, inhibiting TPL2 kinase activity will likely have beneficial effects, such as mitigating the damage from excessive IL-1β. However, TPL2 inhibition could potentiate other inflammatory pathways via elevated type I IFNs. Further exploration of controlled delivery of a TPL2 kinase inhibitor to specific inflammation sites could prove advantageous in inflammatory diseases caused by IL-1β overexpression, such as cryopyrin-associated periodic syndrome and other inflammatory diseases.

## Data Availability

All relevant data is contained within the article. Further inquiries can be directed to the corresponding author/s.
