# Supplementary material for: TPL2 kinase activity is required for Il1b transcription during LPS priming but dispensable for NLRP3 inflammasome activation
Source: Front Immunol. 2025 Mar 18;16:1496613. doi: 10.3389/fimmu.2025.1496613 (PMC11958189; doi:10.3389/fimmu.2025.1496613)
Supplement: Supplementary file 1 [file DataSheet1.docx]

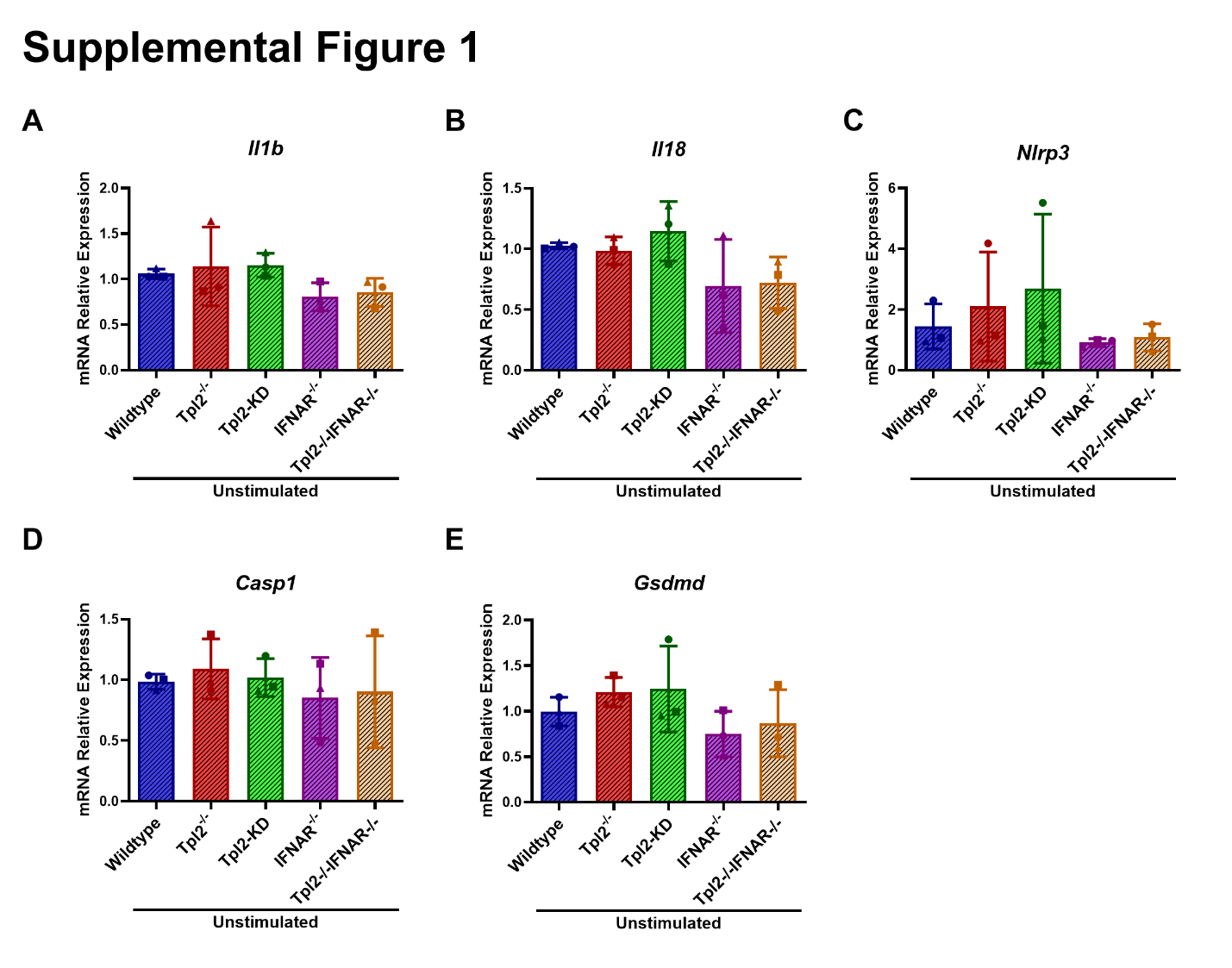


**Supplemental Figure 1**. Unstimulated *Tpl2-KD* and *Tpl2^-/-^* BMDMs have similar mRNA expression levels.

**(A-E)** Unstimulated BMDMs isolated from WT, *Tpl2^-/-^, Tpl2-KD, IFNAR1^-/-^*, and *Tpl2^-/-^IFNAR1^-/-^* BMDMs. Gene expression analysis of *Il1b* **(A)**, *Il18* **(B)**, *Nlrp3* **(C)**, *Casp1* **(D)**, and *Gsdmd* **(E)**.

One-way ANOVA with Tukey’s multiple comparison test was performed. Each data point represents the average of 3 individual mice. Data graphed represent means ± S.E.M. Data are from 3 independent experiments of both male and female mice.


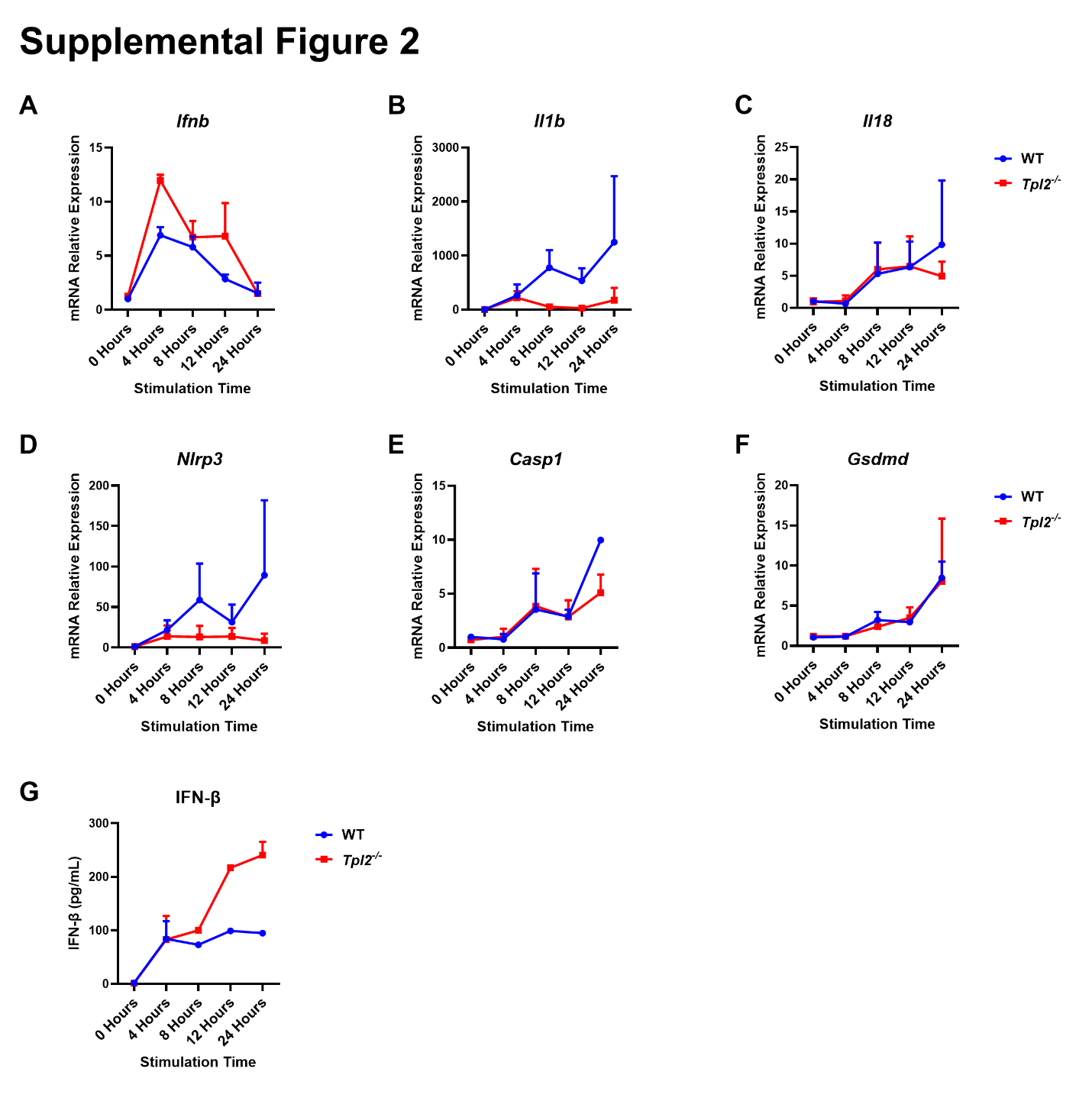


**Supplemental Figure 2**. Expression of inflammasome-processed cytokines and components over 24 hours of LPS stimulation.

**(A-F)** BMDMs isolated from WT and *Tpl2^-/-^* mice were stimulated with 100 ng/mL of LPS over 24 hours. BMDMs were collected for mRNA transcription analysis. Gene expression analysis of *Ifnb* **(A),** *Il1b* **(B)**, *Il18* **(C)**, *Nlrp3* **(D)**, *Casp1* **(E)**, and *Gsdmd* **(F)**.

Each data point represents the average of 2 individual mice. Data graphed represent means ± S.D. Data are from 1 experiment of male mice.

**(G)** BMDMs isolated from WT, *Tpl2^-/-^, IFNAR1^-/-^*, and *Tpl2^-/-^IFNAR1^-/-^* mice were stimulated with 100 ng/mL of LPS over 24 hours. Supernatant was collected to perform an IFN-β ELISA.


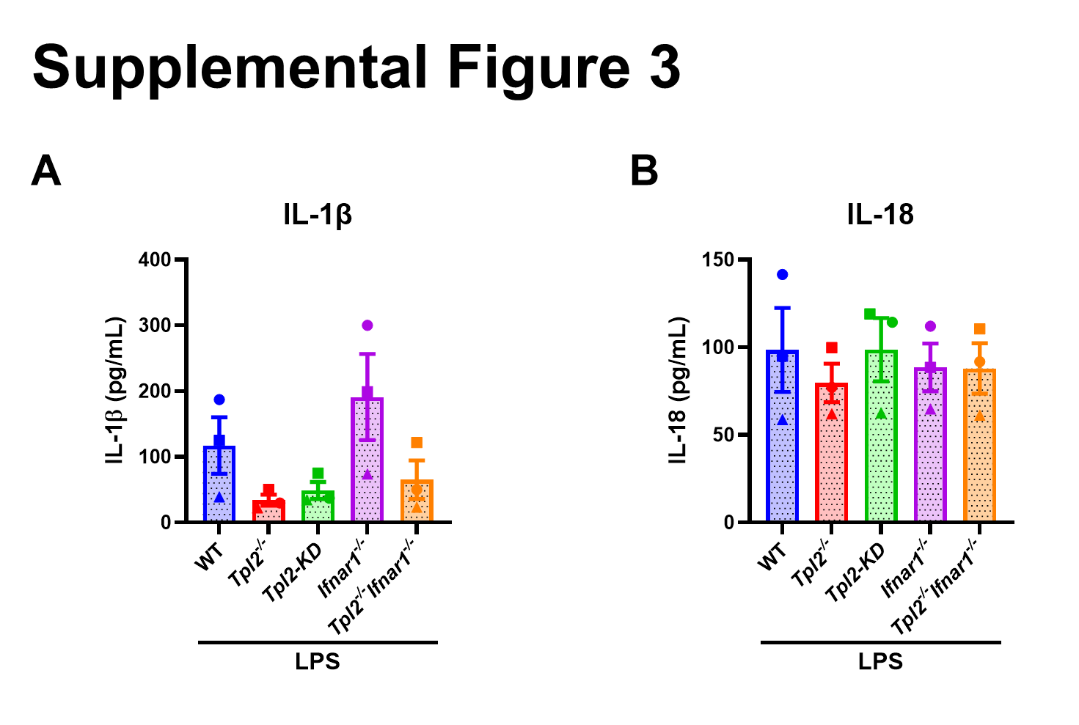


**Supplemental Figure 3**. IL-1β and IL-18 cytokine secretion after 8 hours of LPS stimulation.

**(A and B)** BMDMs isolated from WT, *Tpl2^-/-^, Tpl2-KD, IFNAR1^-/-^*, and *Tpl2^-/-^IFNAR1^-/-^* mice were stimulated with 100 ng/mL of LPS for 4 hours. After 4 hours of LPS stimulation, 5 mM of ATP was added for 4 hours, and supernatant was collected to perform IL-1β and IL-18 ELISAs. Cytokine secretion of IL-1β **(A)** and IL-18 **(B)**.
